# Supplementary material for: An open-source framework for large-scale, flexible evaluation of biomedical text mining systems
Source: J Biomed Discov Collab. 2008 Jan 29;3:1. doi: 10.1186/1747-5333-3-1 (PMC2276192; doi:10.1186/1747-5333-3-1)
Supplement: Additional file 1 — Evaluation platform source code. The additional file contains the software evaluation framework discussed in this paper. The most current release can be downloaded from SourceForge.net [1]. See the accompanying README file for instructions on installation and use. Also included in the distribution is a collection of UIMA wrappers for some commonly used BioNLP tools and annotated corpora. [file 1747-5333-3-1-S1.zip › bionlp_uima_repository-1.0.1/docs/api/edu/uchsc/ccp/uima/util/opendmap/AnnotationString.html]

AnnotationString (Center for Computational Pharmacology Util/UIMA API)


|  |  |  |
| --- | --- | --- |
| **Overview** \| **Package** \| **Class** \| **Tree** \| **Deprecated** \| **Index** \| **Help** | |  |
| **PREV CLASS**   **NEXT CLASS** | **FRAMES**    **NO FRAMES**     **All Classes** |  |
| SUMMARY: NESTED \| FIELD \| CONSTR \| METHOD | DETAIL: FIELD \| CONSTR \| METHOD |  |


---


## edu.uchsc.ccp.uima.util.opendmap Class AnnotationString

```
java.lang.Object
  edu.uchsc.ccp.uima.util.opendmap.AnnotationString
```

---

``` public class AnnotationString extends Object ```

This class generates a string describing a JCas CCPTextAnnotation in a standard
format that can be used for generating and executing regression tests.

**Author:**
:   R. James Firby

---

**Constructor Summary**

`AnnotationString(CCPTextAnnotation annotation,
String[] slots,
String docId,
String docText)`
  
          Create an object that will print as a standard format string for a JCas annotation.


| **Method Summary** | |
| --- | --- |
| `static String` | `create(CCPTextAnnotation annotation, String[] slotNames, String docId, String docText)`             Create a new standard format string for a JCas annotation. |
| `String` | `toString()`             Return the standard format string for this annotation. |

**Methods inherited from class java.lang.Object**

`clone, equals, finalize, getClass, hashCode, notify, notifyAll, wait, wait, wait`

**Constructor Detail**

### AnnotationString

```
public AnnotationString(CCPTextAnnotation annotation,
                        String[] slots,
                        String docId,
                        String docText)
```

:   Create an object that will print as a standard format string for a JCas annotation.

    **Parameters:**: `annotation` - The annotation to describe in the string.: `slots` - The annotation slots to include. Slots with names not in this list are excluded.: `docId` - The ID of the document associated with this annotation: `docText` - The document text associated with this annotatation


**Method Detail**

### toString

```
public String toString()
```

:   Return the standard format string for this annotation.

    :   **Overrides:**: `toString` in class `Object`

---


### create

```
public static String create(CCPTextAnnotation annotation,
                            String[] slotNames,
                            String docId,
                            String docText)
```

:   Create a new standard format string for a JCas annotation.

    :   **Parameters:**: `annotation` - The annotation to describe in the string.: `slots` - The annotation slots to include. Slots with names not in this list are excluded.: `docId` - The ID of the document associated with this annotation: `docText` - The document text associated with this annotatation


---


|  |  |  |
| --- | --- | --- |
| **Overview** \| **Package** \| **Class** \| **Tree** \| **Deprecated** \| **Index** \| **Help** | |  |
| **PREV CLASS**   **NEXT CLASS** | **FRAMES**    **NO FRAMES**     **All Classes** |  |
| SUMMARY: NESTED \| FIELD \| CONSTR \| METHOD | DETAIL: FIELD \| CONSTR \| METHOD |  |


---
